# Nodular secondary syphilis in a patient who was immunocompetent: a sporotrichoid masquerader with synchronous primary lesion

**DOI:** 10.1093/skinhd/vzag026

**Published:** 2026-04-17

**Authors:** Jacopo Tartaglia, Francesco Fortarezza, Monica Ponzano, Angelo Paolo Dei Tos, Mauro Alaibac

**Affiliations:** Dermatology Unit, Department of Medicine (DIMED), University of Padova, Padova, Italy; Surgical Pathology and Cytopathology Unit, University Hospital of Padova, Padova, Italy; Dermatology Unit, Department of Medicine (DIMED), University of Padova, Padova, Italy; Surgical Pathology and Cytopathology Unit, University Hospital of Padova, Padova, Italy; Dermatology Unit, Department of Medicine (DIMED), University of Padova, Padova, Italy

## Abstract

We report a case of nodular secondary syphilis in an immunocompetent man presenting with concurrent chancre, macular rash and sporotrichoid nodules. Dermoscopy and histology raised suspicion for pseudolymphoma, but serology and Warthin–Starry staining confirmed the diagnosis.

Dear Editor, Nodular secondary syphilis is an uncommon clinical form of *Treponema pallidum* infection, traditionally described in patients who are immunocompromised, but it is also seen in people who are immunocompetent.^1,2^ Its clinical presentation may resemble deep fungal infections, atypical mycobacterioses, granulomatous dermatoses or cutaneous lymphomas. Although the primary and secondary stages of syphilis usually follow a chronological sequence, both may coexist in up to 40% of cases, which may lead to diagnostic uncertainty when lesions of different stages are present simultaneously.^3^

We present the case of a man who was immunocompetent with coexisting primary and secondary lesions, in whom nodular secondary syphilis mimicked sporotrichosis clinically, while histopathology raised suspicion for cutaneous pseudolymphoma. The patient was considered to be immunocompetent, as HIV serology was negative, there was no history of having taken immunosuppressive medication, there was no history of autoimmune or haematological disorders, and there was no clinical or laboratory evidence of an underlying malignancy. In the absence of systemic symptoms, lymphadenopathy or laboratory abnormalities suggestive of lymphoma, no additional radiology was performed to screen for an occult malignancy. He was employed in the logistics sector within a healthcare facility, with no relevant occupational exposures.

The patient presented with a painless genital ulcer that had persisted for 6 weeks, followed by the development of multiple erythematous macules on the trunk and firm red nodules on the right arm. Examination revealed a 1-cm chancre on the frenulum (Figure 1a, b), scattered roseola-like macules on the trunk (Figure 1c) and multiple firm, dome-shaped nodules on the right upper limb, some in a linear (sporotrichoid) distribution (Figure 2a, b). One nodule on the hand exhibited a central hyperkeratotic crust with a fissure (Figure 2a).

**Figure 1:**
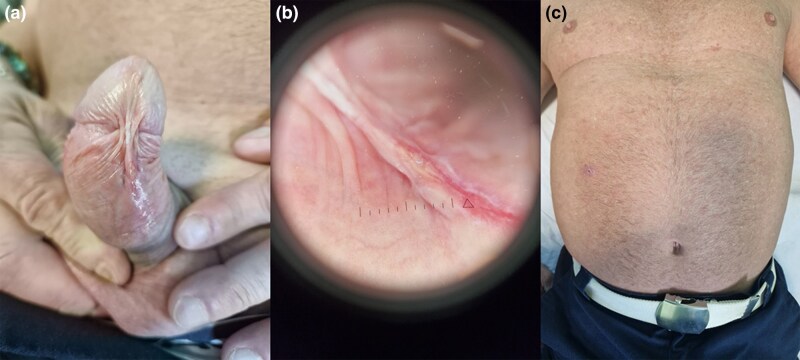
(a) Primary chancre on the frenulum: well-demarcated, approximately 1-cm ulcer with a moist base and indurated border. (b) Dermoscopic appearance of the frenular ulcer, showing central erosion, dotted vessels and surrounding erythema (diameter approximately 1 cm). (c) Roseola-like rash of secondary syphilis: multiple faint, erythematous, nondesquamating macules scattered over the chest, abdomen and back.

**Figure 2:**
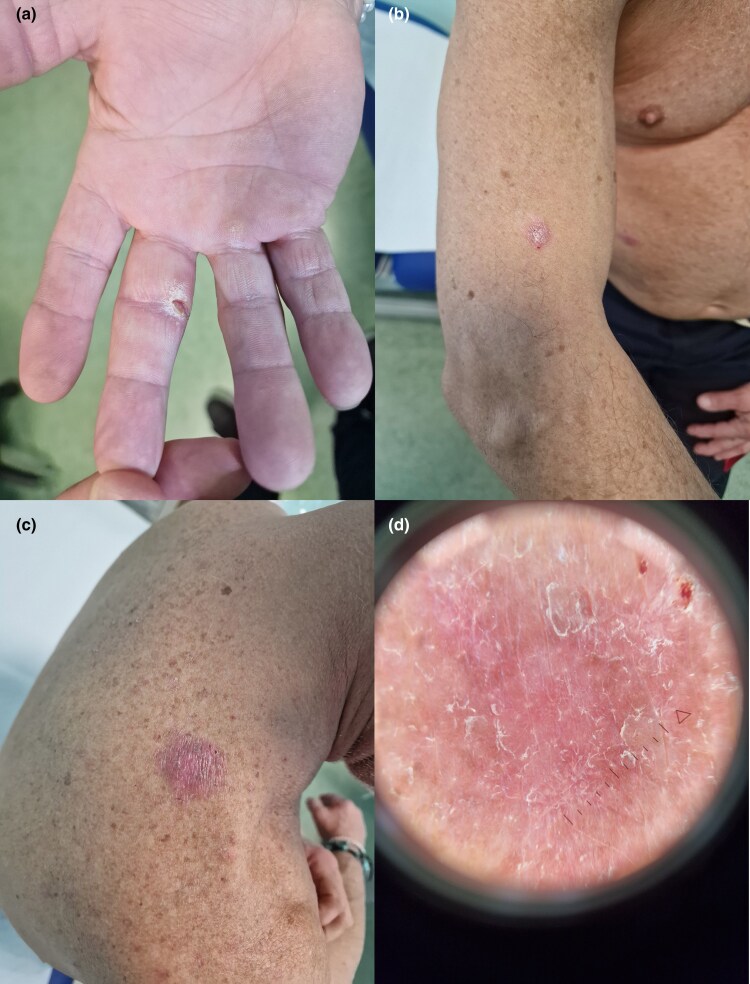
Sporotrichoid-like cutaneous lesions of the right upper limb. (a–c) Multiple erythematous papules and nodules distributed in a linear pattern from the hand to the shoulder. (a) One nodule on the hand displays a hyperkeratotic crust with a central fissure. (d) Dermoscopic appearance of a dominant erythematous nodule on the right shoulder: structureless pink background with diffuse white scaling, more pronounced at the periphery. Dotted vessels and interlacing white lines are also evident.

Dermoscopic examination of a dominant erythematous nodule on the right shoulder revealed a structureless pink background with diffuse white scaling, more prominent towards the periphery of the lesion (Figure 2c). Dotted vessels and interlacing white areas were also seen.

A 6-mm punch biopsy was obtained from the dominant right shoulder nodule. Histopathological examination revealed a dense dermal inflammatory infiltrate with a prominent plasmacytic component, arranged in a perivascular distribution throughout the dermis (Figure 3). The overlying epidermis was unaffected and separated from the infiltrate by a grenz zone of preserved collagen. No granulomas were identified. Polymerase chain reaction testing for atypical mycobacteria and Ziehl–Neelsen staining were both negative. Spiral-shaped bacilli morphologically consistent with *Treponema* spp. were identified around basal keratinocytes in the epidermis by Warthin–Starry stain. The final diagnosis was cutaneous pseudolymphoma with a prominent plasmacellular component, and clinical investigation for an underlying treponemal infection was recommended. Serological testing for syphilis was performed, with a positive *T. pallidum* haemagglutination assay and a Venereal Disease Research Laboratory (VDRL) test (titre 1:128).

**Figure 3:**
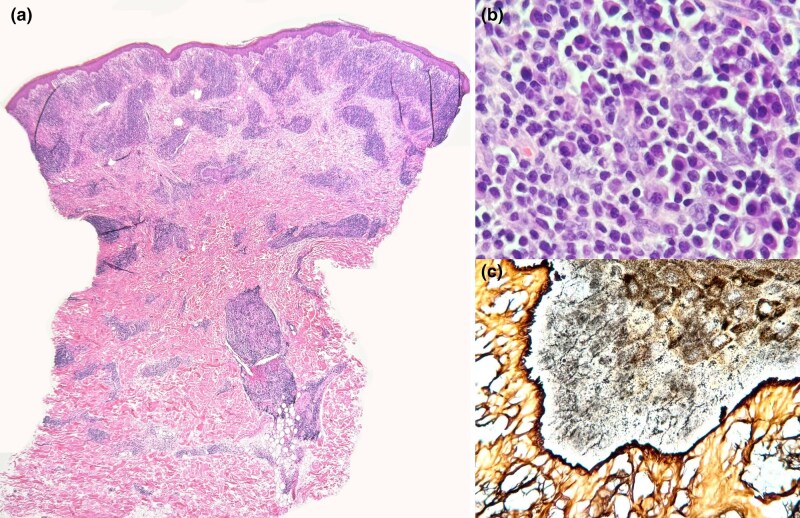
(a) Panoramic view of a skin punch biopsy showing a dense perivascular and periadnexal lymphoid infiltrate involving the full thickness of the dermis, with a subtle grenz zone (haematoxylin and eosin, original magnification ×2.5). (b) At higher magnification, the infiltrate is predominantly composed of plasma cells (haematoxylin and eosin, original magnification ×200). (c) Warthin–Starry stain highlights spiral-shaped bacilli within the basal and parabasal layers of the epidermis (Warthin–Starry stain, original magnification ×200).

The patient was treated with benzathine penicillin G, 2.4 million units intramuscularly in a single dose. Over the next few months, the nodules and rash completely resolved. At the 6-month ­follow-up, the VDRL titre had declined fourfold.

Nodular secondary syphilis is a rare presentation of *T. pallidum* infection, classically associated with immunosuppression but increasingly reported in people who are immunocompetent.^4^ In our case, the linear nodular spread suggested sporotrichoid infection, while histology revealed a dense lymphoplasmacytic infiltrate with a grenz zone, raising suspicion for pseudolymphoma. Similar histological features have been reported in nodular syphilis, with or without granulomas.^5,6^

Notably, our patient exhibited concurrent chancre, roseola-­like macules and nodular lesions, an uncommon but documented overlap of primary and secondary stages, occurring in up to 40% of cases.^3^ This overlap may complicate diagnosis if only one lesion type is sampled or recognized. Dermoscopy, although rarely described in syphilis, revealed dotted vessels, interlacing white lines and peripheral collarette scaling resembling Biett sign. In our patient, interlacing white lines and dotted vessels were also seen.

The recommended first-line treatment for early syphilis, including secondary forms, is a single intramuscular dose of benzathine penicillin G (2.4 million units), as stated in the 2021 Centers for Disease Control Sexually Transmitted Infections Treatment Guidelines.^7^ No specific modifications are currently recommended for nodular or pseudolymphomatous variants. Published case reports and small series of nodular secondary syphilis consistently describe benzathine penicillin G as the first-line therapy, most often administered either as a single injection or as three weekly doses, with complete or near-complete resolution of lesions across regimens.^8,9^ In addition, a systematic review of 45 cases of malignant (ulceronodular) secondary syphilis showed that approximately two-thirds of patients were treated with intramuscular benzathine penicillin G, while the remaining patients received intravenous aqueous penicillin G, doxycycline or ceftriaxone, and all experienced a rapid clinical improvement after antibiotic therapy.^10^ These data support the notion that even nodular and ulceronecrotic forms are highly responsive to standard antitreponemal regimens and do not necessarily require intensification beyond guideline-based schedules when late latent disease and neurosyphilis have been excluded. Our patient received a single benzathine penicillin G injection, and showed rapid resolution of lesions. At the 3-month follow-up, complete clinical clearance was documented; at 6 months, the VDRL titre had declined fourfold, consistent with the expected serological response after appropriate therapy.
